# Associations between Vitamin D Status and Type 2 Diabetes Measures among Inuit in Greenland May Be Affected by Other Factors

**DOI:** 10.1371/journal.pone.0152763

**Published:** 2016-04-13

**Authors:** Nina O. Nielsen, Peter Bjerregaard, Pernille F. Rønn, Henrik Friis, Stig Andersen, Mads Melbye, Marika Lundqvist, Arieh S. Cohen, David M. Hougaard, Marit E. Jørgensen

**Affiliations:** 1 National Institute of Public Health, University of Southern Denmark, Copenhagen, Denmark; 2 Greeenland Centre for Health Research, University of Greenland, Nuuk, Greenland; 3 Steno Diabetes Centre, Gentofte, Denmark; 4 Department of Public Health, Aarhus University, Aarhus, Denmark; 5 Department of Nutrition, Exercise and Sports, University of Copenhagen, Copenhagen, Denmark; 6 Aalborg University Hospital, Aalborg, Denmark; 7 Department of Epidemiology Research, Statens Serum Institut, Copenhagen, Denmark; 8 Department of Congenital Disorders, Statens Serum Institut, Copenhagen, Denmark; College of Tropical Agriculture and Human Resources, University of Hawaii, UNITED STATES

## Abstract

**Objective:**

Epidemiological studies have provided evidence of an association between vitamin D insufficiency and type 2 diabetes. Vitamin D levels have decreased among Inuit in Greenland, and type 2 diabetes is increasing. We hypothesized that the decline in vitamin D could have contributed to the increase in type 2 diabetes, and therefore investigated associations between serum 25(OH)D3 as a measure of vitamin D status and glucose homeostasis and glucose intolerance in an adult Inuit population.

**Methods:**

2877 Inuit (≥18 years) randomly selected for participation in the Inuit Health in Transition study were included. Fasting- and 2hour plasma glucose and insulin, C-peptide and HbA_1c_ were measured, and associations with serum 25(OH)D3 were analysed using linear and logistic regression. A subsample of 330 individuals who also donated a blood sample in 1987, were furthermore included.

**Results:**

After adjustment, increasing serum 25(OH)D3 (per 10 nmol/L) was associated with higher fasting plasma glucose (0.02 mmol/L, p = 0.004), 2hour plasma glucose (0.05 nmol/L, p = 0.002) and HbA_1c_ (0.39%, p<0.001), and with lower beta-cell function (-1.00 mmol/L, p<0.001). Serum 25(OH)D3 was positively associated with impaired fasting glycaemia (OR: 1.08, p = 0.001), but not with IGT or type 2 diabetes.

**Conclusions:**

Our results did not support an association between low vitamin D levels and risk of type 2 diabetes. Instead, we found weak positive associations between vitamin D levels and fasting- and 2hour plasma glucose levels, HbA_1c_ and impaired fasting glycaemia, and a negative association with beta-cell function, underlining the need for determination of the causal relationship.

## Introduction

Previously, low vitamin D was mainly a concern in relation to calcium homeostasis, and deficiency could cause the bone-deforming rickets disease in children and lead to the development of osteoporosis and osteomalacia in adults. However, during the last decades epidemiological evidence from various parts of the world has linked low vitamin D levels with metabolic and cardiovascular disorders, infectious and inflammatory diseases, and some cancer types [1–2], indicating a broader role of vitamin D in human health. Associations between low vitamin D status and type 2 diabetes have been reported in several cross-sectional studies [3, 4], as well as prospective cohort studies [5–7]. Two recent large prospective cohort studies provided evidence of an inverse association between serum 25-hydroxyvitamin D (25(OH)D) and markers of glucose homeostasis [6] and risk of type 2 diabetes [7], indicating a causal relationship. In contrast, some studies have reported lack of association between 25(OH)D levels and incident type 2 diabetes [8], and Mendelian randomization studies and randomized placebo-controlled trials have questioned a causal association between vitamin D and type 2 diabetes [9, 10]. Different methods and designs have been used in these studies. Some have measured the dietary intake of vitamin D, which does not account for the large proportion synthesized in the skin [11, 12], whereas others have used blood 25-hydroxyvitamin D (25(OH)D) as a biomarker of vitamin D status [8]. Furthermore, the recording of diabetes varies among self-report [8], medication registry data [13] and diagnosis based on blood glucose levels [6].

In most areas of the world dermal production of vitamin D from exposure to sunlight is the main source of vitamin D. However, high latitude populations (above 40°N) are incapable of producing sufficient amounts of vitamin D in the winter months, and during this period of the year diet is the main source. As part of the Arctic region, Greenland is located at very high latitudes (60°N–78°N). The Inuit population therefore experiences extended periods of darkness, and even in the periods where sunlight is present, vitamin D synthesis is limited due to the high solar zenith angle [14] and the continued need for outdoor clothing [15]. Consequently, dietary vitamin D is crucial to this population. Previously, the traditional diet containing fish and sea mammals probably maintained a healthy vitamin D status among the Inuit [16]. However, as a result of the extensive nutrition transition which has occurred in Greenland during the past 50–60 years, the traditional diet has to a large extent been substituted by imported foods such as fatty meat, sweets, chips, cakes and soft drinks. We recently reported a profound decrease in vitamin D status from 1987 to 2010, due to a change away from a traditional diet [17].

In this study, we aimed to examine the association between vitamin D and glucose homeostasis and glucose intolerance in the same Inuit population. Our study population is particularly appropriate for this purpose due to the low vitamin D status and increasing prevalence of type 2 diabetes; the current prevalence is 7–10% among adults [18, 19]. Furthermore, we have a subsample of the study population with data on vitamin D status in 1987, which allows analyses of associations between former vitamin D status and later development of type 2 diabetes. We hypothesised that low vitamin D levels were associated with higher risk of type 2 diabetes. Serum 25-hydroxyvitamin D3 (25(OH)D3) concentration was used as a measure of vitamin D status, and HbA_1c_ and fasting- and 2hour plasma glucose and insulin levels were used as measures of glucose homeostasis and glucose intolerance.

## Methods

### Study participants and design

The total population of Greenland is 57.000, and approximately 90% of the population is indigenous Greenlanders (Inuit), while the remaining are mainly Danes. The population inhabits 16 towns and approximately 60 villages. Study participants were part of a general health study among adults (all age groups ≥18 years) in Greenland established in 2005–2010 (Inuit Health in Transition (IHIT) study) [20]. Participants in the main health study were selected through a random sampling of adults with permanent residence in Greenland. Greenland was divided into twelve regions, and from each region a number of villages and towns were chosen to be included in the study. Twenty-two communities, including the capital Nuuk, eight smaller towns, and 13 villages were selected as study areas being representative of each region in Greenland [20, 21]. In villages, all adult inhabitants were invited to participate, whereas in towns, a random sample of 11–22% was drawn from the central population register. With a participation rate for Inuit of 68%, the total study sample consisted of 3108 Inuit (9.1% of the total adult population in 2005) representing all geographical areas and community sizes. Categorization as Inuit was based on the primary language and self-identification. From 2877 (93%), a serum 25(OH)D3 measurement was obtained, which was included in this cross-sectional study. Before analyses of associations, non-fasting individuals and individuals with previously diagnosed diabetes were excluded (n = 144). Among individuals with a serum 25(OH)D3 measurement, 485 individuals had donated a blood sample as part of their participation in a population-based serological syphilis survey carried out in 1987 in western and southern districts of Greenland [22]. The syphilis screening, and treatment, was carried out following an epidemic in April 1987 and was offered to all individuals aged 15–60 years; the 485 individuals represented inhabitants of Aasiaat, Maniitsoq, Qaqortoq, and Narsaq. From 330 of these, a blood sample was stored and available for measurement of 25(OH)D3. These historical samples allowed the assessment of associations between past serum 25(OH)D3 levels and future glucose metabolism.

### Collection and handling of blood samples

Blood samples from 1987 were drawn from May to June, whereas samples from 2005–2010 were collected during all months of the year in the period April 2005 to October 2010, except for July, November and December (due to annual leave and inaccessibility). Samples from 2005–2010 were collected as part of the IHIT study in nine towns and 13 villages by expeditions along the north-west, south and the east coasts of Greenland. Blood samples were handled and stored as described elsewhere [17].

### Insulin and glucose measures

Fasting blood samples were drawn from individuals who had spent a minimum of eight hours without consuming any food or liquids. Participants underwent a 2hour OGTT, in which they received 75 g of glucose, and another blood sample was taken. Plasma glucose was analyzed using Hexokinase/G6P-DH-Determiation on a Hitachi 912 System. Insulin measures were analyzed by two-site fluoroimmunometric assay for quantification of intact insulin in human serum (Wallac Auto Delfia). C-peptide was measured in pmol/L in serum, which was allowed to rest for 30–150 minutes before centrifugation at 20°C, 3000 rpm for 10 minutes. After centrifugation, the C-peptide samples were frozen at -20°C. C-peptide was analysed by Immunoassay for the in vitro quantitative determination of C-peptide in human plasma using Cobas e411, Roche. HbA_1c_ was taken in a capillary tube and analyzed by the principles of an ion-exchange HPLC Bio-Rad variant (Hercules, California) as described elsewhere [23]. Samples were kept refrigerated until analysis of HbA_1_c. All measurements were performed at the Steno Diabetes Centre, Gentofte, Denmark. Hepatic insulin resistance (HOMA-IR) was calculated as fasting insulin (pmol/L) * fasting plasma glucose (mmol/L)/22.5. The Insulin sensitivity index (ISI_0,120_) was calculated according to Gutt el al. [24] to provide an estimate of insulin sensitivity in the peripheral tissues as follows:

$$\begin{array}{l} {\text{ISI}}_{0,120}=[\underline{75,000+(\text{fasting glucose}\times 18\;\text{-}\;2\text{hour glucose}\times 18)\times 0.19\times \text{body weight}\;\left(\text{kg}\right)]∕120\;\text{min}}\\ \begin{array}{l} \left(\text{fasting glucose}+2\text{hour glucose}\right)∕2\\ ∕\text{log}\;\left(\text{fasting insulin}∕6.945\right)+\left(2\text{hour insulin}∕6.945\right)∕2 \end{array} \end{array}$$

As a measure of beta-cell function the fasting C-peptide/fasting insulin ratio was calculated. According to the current WHO-guidelines [25], impaired fasting glycaemia (IFG), impaired glucose tolerance (IGT), and type 2 diabetes were defined as follows: IFG = fasting plasma glucose 6.1 to 6.9 mmol/L and 2hour plasma glucose < 7.8 mmol/L, IGT = fasting plasma glucose < 7.0 mmol/L and 2hour plasma glucose ≥ 7.8 and < 11.1 mmol/L, type 2 diabetes = fasting plasma glucose ≥ 7.0 mmol/L or 2hour plasma glucose ≥ 11.1 mmol/L, and/or a history of type 2 diabetes.

### Vitamin D measurements

IHIT samples were stored for 3–7 years and the 1987-samples for 26 years at -80°C before thawn and analyzed for 25(OH)D3 and 25(OH)D2. Analyses were performed by liquid chromatography-tandem mass spectrometry (LC-MSMS) using the “MSMS vitamin D” kit from Perkin Elmer (Waltham, MA) as described previously [26]. This method measured both serum 25(OH)D3 and 25(OH)D2, but due to the negligible 25(OH)D2 concentrations detected, only 25(OH)D3 was considered in the analyses.

### Potential confounders

In connection with blood sample collections in the IHIT study, information on potential confounders was obtained. Data on ancestry (fully or partly Inuit), smoking, physical activity energy expenditure, and use of supplements (vitamin D and multivitamins) were collected by interview-guided questionnaires. Ancestry was based on the grandparent’s ethnicity. A participant with four Inuit grandparents was defined as fully Inuit, whereas a participant with 1–3 grandparents of non-Inuit descent was defined as partly Inuit. Physical activity was recorded by the International Physical Activity Questionnaire (long version IPAQ) validated and modified for the living conditions of the Greenlandic population [27]. Anthropometric measurements were used to calculate body mass index (BMI) as: ((weight in kg)/(height in m)^2^). Data on total blood mercury levels were obtained from measurements in serum samples. Information on potential confounders beyond age and sex was not available from the 1987-sample.

### Ethics

Participants in the IHIT-study gave their informed written consent to participate in health investigations at inclusion in the study. The present study, including use of the stored samples from 1987, was reviewed and approved by the Ethical Review Committee for Greenland. The samples from 1987 are from a population-based serological syphilis survey [21] and were stored at The Danish National Biobank, Statens Serum Institut, Copenhagen, Denmark (http://www.ssi.dk/English/Service/AboutSSI/Organization/Organisationchart/Department.aspx?id=e4091b0d-0269-4445-9fe5-9db500a0483e) from where they were procured. Since all study participants gave their informed written consent to participate in the IHIT-study in 2005–2010, the Ethical Review Committee for Greenland waived the need for consent regarding the samples previously collected from the same individuals in 1987.

### Statistical methods

The statistical analyses were performed in STATA 12 and SAS 9.3 (SAS Institute Inc., Carey, NC, USA). All continuous outcome variables, except fasting plasma glucose and 2hour plasma glucose, were transformed using the natural logarithm function to obtain normal distributions before entered in linear regression models. Transformed coefficients were back-transformed and expressed as percentage change per 10 nmol/L increase in serum 25(OH)D3. Associations between serum 25(OH)D3 levels and fasting plasma glucose and 2hour plasma glucose were expressed as change in units (mmol/L) per 10 nmol/L increase in serum 25(OH)D3. We tested for non-linearity by inclusion of a quadratic term of serum 25(OH)D3. Since a borderline significant non-linear relation was observed between 25(OH)D3 and HbA_1c_, we used multiple regression with quadratic splines to visually investigate the relation, with 25(OH)D3 as explanatory variable and HbA_1c_ as outcome variable. The spline was interpolated with four knots placed at 40 nmol/L, 80 nmol/L, 120 nmol/L and 160 nmol/L on the 25(OH)D3 scale. We predicted values for a man with prespecified values of the covariates (44 years, smoker, fully Inuit, BMI of 26 kg/m^2^ and physical activity energy expenditure of 46 kJ/kg/day). We tested for linearity by comparing the spline model with a model with linear effect of 25(OH)D3.

Associations between serum 25(OH)D3 and categorical outcome variables (IFG, IGT and type 2 diabetes) were assessed by logistic regression models.

## Results

### Characteristics of the study population

Of the 330 participants in the 1987-sample and the 2877 individuals in the IHIT study (2005–2010) with data on serum 25(OH)D3, 319 (97%) and 2566 (89%), respectively, had complete data on all outcome variables and selected confounders. The median age (interquartile range, IQR) of the 1987-sample and the IHIT-sample was 31 (IQR: 23–40) years and 44 (IQR: 34–54) years, respectively. The median (IQR) serum 25(OH)D3 was 59.0 (IQR: 38.3–90.5) nmol/L in the 1987-sample and 46.6 (IQR: 29.7–67.4) nmol/L in the IHIT-sample, and the respective proportions of men in the two study populations were 41% and 44%.

Table 1 gives characteristics of the study population in 1987 and 2005–2010 by sex. Median serum 25(OH)D3 was higher among men than women in 1987 (64 vs. 57 nmol/L), but did not differ between men and women in 2005–2010 (49 vs. 45 nmol/L). Median BMI was remarkably high among both men and women, but higher among women (25 vs. 26 kg/m^2^), whereas the level of physical activity energy expenditure (52 vs. 46 kJ/kg/day) and total blood mercury (19 vs. 14 μg/L) were higher among men. Fasting plasma glucose and HbA_1c_ were also higher among men, whereas 2hour plasma glucose and fasting- and 2hour insulin were higher among women. Overall, the prevalence of IFG, IGT, and type 2 diabetes was 13%, 5.3% and 8.8%, respectively. IFG was more prevalent among men than women (16% vs. 11%), whereas IGT was more prevalent among women than men (6.8% vs. 3.2%). The prevalence of type 2 diabetes was not different among men and women (10% vs. 8%).

**Table 1:** Characteristics of the study population in 1987 (N = 330) and 2005–2010 (N = 2877).

**1987**	**n**	**Men**	**n**	**Women**
Age (years)	134	33 (24–41)	196	29 (22–39)
25(OH)D3 (nmol/L)*	134	64 (39–103)	196	57 (36–82)
**2005–2010**	**n**	**Men**	**n**	**Women**
Age (years)	1256	45 (35–55)	1621	43 (33–53)
BMI (kg/m^2^)	1241	25 (22–29)	1609	26 (23–30)
25(OH)D3 (nmol/L) ^†^	1256	49 (29–70)	1621	45 (30–66)
Physical activity energy expenditure (kJ/kg/day)	1254	52 (23–97)	1620	46 (24–74)
Fasting plasma glucose (mmol/L)	1242	5.7 (5.3–6.1)	1606	5.5 (5.2–5.9)
2hour plasma glucose (mmol/L)	1202	5.0 (4.0–6.4)	1545	5.7 (4.7–7.0)
Fasting insulin (pmol/L)	1243	33 (22–50)	1606	42 (29–61)
2hour insulin (pmol/L)	1202	63 (30–142)	1544	148 (80–249)
HbA_1C_ (mmol/mol)	1250	39 (37–41)	1617	38 (36–41)
HbA_1C_ (%)	1250	5.7 (5.5–5.9)	1617	5.6 (5.4–5.9)
Mercury (μg/L)	1254	19 (8.2–42)	1617	14 (6.6–32)
Supplementation^‡^	1256	8.2 (103)	1621	12.8 (207)
Ancestry, % fully Inuit (n)	1241	86 (1071)	1600	88 (1407)
Smokers, % (n)	1254	66 (825)	1617	69 (1123)
IFG, % (n)	1217	16 (189)	1566	11 (169)
IGT, % (n)	1217	3.2 (39)	1566	6.8 (107)
IFG+IGT, % (n)	1217	3.0 (36)	1566	3.5 (55)
Type 2 diabetes, % (n)	1217	10 (116)	1566	8 (128)

Data are median (interquartile range) unless otherwise stated.

* Median concentration in May-June 1987.

^†^ Median concentration in all months except July, November and December 2005–2010.

^‡^Vitamin D or multivitamins.

### Associations with glucose homeostasis

Overall, there was no interaction between sex and the association between serum 25(OH)D3 and markers of glucose homeostasis. Analyses were therefore performed for men and women together.

Table 2 shows the association between serum 25(OH)D3 measured in the IHIT-study in 2005–2010 and markers of glucose homeostasis measured in the same investigation before and after adjustment for potential confounders. After adjustment for sex, age, ancestry, BMI, smoking, physical activity, and supplementation with vitamin D or multivitamins (Model 2), increasing 25(OH)D3, expressed per 10 nmol/L, was associated with increasing fasting plasma glucose (0.03 mmol/L, 95% CI: 0.02; 0.04), 2hour plasma glucose (0.06 mmol/L, CI: 0.03; 0.09) and HbA_1c_ (0.51%, 95% CI: 0.37%; 0.66%). In contrast, increasing 25(OH)D3 levels were associated with decreasing beta-cell function (-0.82%, CI: -1.30; -0.33). No association with peripheral insulin sensitivity (ISI_0,120_) and HOMA-IR was found after adjustment for confounders. In Model 3, the associations between 25(OH)D3 and glucose homeostasis measures were additionally adjusted for total blood methyl mercury concentrations after assessment of sufficient variation in the relation between 25(OH)D3 and mercury. After full adjustment, the weak positive association with fasting plasma glucose (0.02 mmol/L; CI: 0.01; 0.03), 2hour plasma glucose (0.05 mmol/L, CI: 0.02; 0.08) and HbA_1c_ (0.39%, 95% CI: 0.23%; 0.54%) remained significant, and the inverse association with beta-cell function also persisted (-1.00%; CI: -1.52; -0.47).

**Table 2:** Association between serum 25(OH)D3 (nmol/L) and measures of glucose homeostasis in 2005–2010.

	Model 1			Model 2			Model 3		
	Unadjusted model			Adjusted for sex, age, ancestry (Inuit or partly Inuit), BMI, smoking, physical activity, and supplementation*			Additionally adjusted for total blood mercury		
	n	Change (95% CI)	P-value	n	Change (95% CI)	P-value	n	Change (95% CI)	P-value
**Association with 25(OH)D3**									
Fasting plasma glucose	2733	0.11 (0.08; 0.14)	<0.001	2580	0.03 (0.02; 0.04)	<0.001	2577	0.02 (0.01; 0.03)	0.004
2hour plasma glucose	2720	0.23 (0.14; 0.31)	<0.001	2570	0.06 (0.03; 0.09)	<0.001	2567	0.05 (0.02; 0.08)	0.002
Peripheral insulin sensitivity (ISI _0,120_)	2693	-3.92 (-5.84; -1.96)	<0.001	2569	-0.24 (-0.90; 0.43)	0.49	2566	-0.38 (-1.10; 0.34)	0.30
Fasting c-peptide/fasting insulin ratio	2733	0.82 (0.33; 1.31)	0.001	2580	-0.82 (-1.30; -0.33)	0.001	2577	-1.00 (-1.52; -0.47)	<0.001
HOMA-IR	2733	0.93 (-1.42; 3.33)	0.44	2580	-0.37 (-1.10; 0.36)	0.33	2577	-0.34 (-1.12; 0.46)	0.41
HbA_1c_	2723	2.08 (1.67; 2.49)	<0.001	2570	0.51 (0.37; 0.66)	<0.001	2567	0.39 (0.23; 0.54)	<0.001

Associations are expressed as change in units (for fasting plasma glucose and 2hour plasma glucose) or percent change (for ln-transformed outcomes) with 95% confidence intervals per 10 nmol/L increase in 25(OH)D3. Only fasting individuals with newly diagnosed glucose intolerance were included in the analyses.

*Vitamin D or multivitamins.

**Fig 1:**
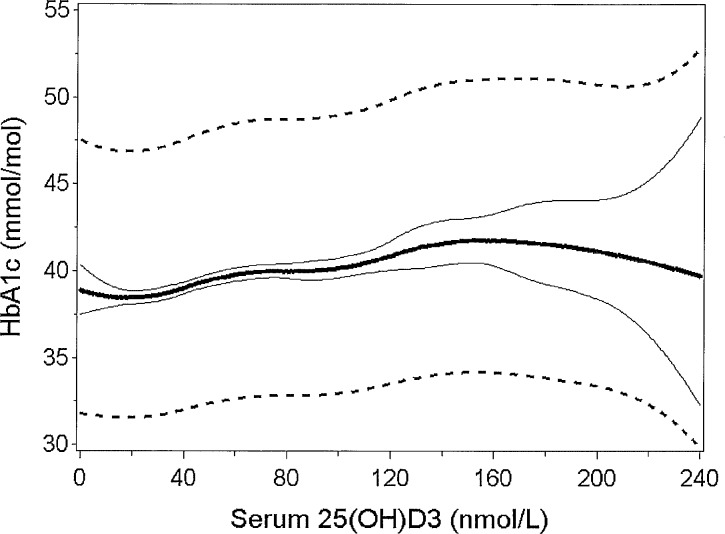
Relation between serum 25(OH)D3 and HbA1c. Quadratic spline analysis showing the relation between serum 25(OH)D3 concentrations and HbA1c for a man aged 44 years, being full Inuit and smoker, and with a BMI of 26 kg/m^2^ and a physical activity energy expenditure of 46 kJ/kg/day. The thick line represents the relation predicted, the full thin lines show the 95% confidence interval, and dotted lines show the 95% prediction interval. N = 2355.

Due to a borderline significant quadratic term of serum 25(OH)D3 in relation to HbA_1c_, indicating a non-linear association, we investigated this association further with spline analyses (Fig 1). The shape of the association between 25(OH)D3 and HbA_1c_ did not vary much from a linear association, and a statistical test likewise indicated no deviation from linearity (p = 0.26).

When using 25(OH)D3 levels measured in samples from 1987 as a predictor of the glucose homeostasis outcomes measured in 2005–2010, increasing 25(OH)D3 concentrations were associated with increasing HbA_1c_ (0.46%, 95% CI: 0.26%; 0.65%) and peripheral insulin sensitivity (2.09%, 95% CI: 0.26%; 3.96%) after adjustment for age and sex, whereas no associations with fasting- and 2hour plasma glucose, beta-cell function and HOMA-IR, were found.

### Associations with glucose intolerance

Table 3 gives the unadjusted and adjusted associations between serum 25(OH)D3 and IFG, IGT and type 2 diabetes in 2005–2010. In Model 1 and 2 the estimates indicated a positive association with IFG and type 2 diabetes. For each 10 nmol/L increase in serum 25(OH)D3, the adjusted odds of IFG increased by 10% (OR: 1.10, CI: 1.06; 1.14), and by 7% (OR: 1.07, CI: 1.01; 1.12) for type 2 diabetes after adjustment for confounders, whereas no association with IGT was observed. In Model 3, the associations with IFG remained significant (OR: 1.08, CI: 1.03; 1.12), whereas the association with type 2 diabetes disappeared after additional adjustment for mercury. Serum 25(OH)D3 measured in 1987 was not associated with IFG (OR: 1.08, CI: 0.99; 1.17; n = 260), IGT (OR: 0.99, CI: 0.86; 1.13; n = 241) or type 2 diabetes (OR: 0.99, CI: 0.88; 1.11; n = 321) as defined in 2005–2010 after adjustment for age and sex. The number of cases with IFG, IGT and type 2 diabetes among the participants with a serum 25(OH)D3 measurement from 1987 was 41 (12.4%), 22 (6.7%), 33 (10.0%), respectively.

**Table 3:** Association between serum 25(OH)D3 (nmol/L) and glucose intolerance in 2005–2010.

	Model 1			Model 2			Model 3		
	Unadjusted model			Adjusted for sex, age, ancestry (Inuit or partly Inuit), BMI, smoking, physical activity, and supplementation^§^			Additionally adjusted for total blood mercury		
	n	OR (95% CI)	P-value	n	OR (95% CI)	P-value	n	OR (95% CI)	P-value
**Association with 25(OH)D3**									
IFG*	2302	1.16 (1.12; 1.19)	<0.001	2175	1.10 (1.06; 1.14)	<0.001	2172	1.08 (1.03; 1.12)	0.001
IGT^†^	2090	1.06 (1.01; 1.11)	0.03	1979	0.98 (0.91; 1.05)	0.62	1977	0.98 (0.91; 1.06)	0.63
Type 2 diabetes^‡^	2733	1.30 (1.15; 1.45)	<0.001	2580	1.07 (1.01; 1.12)	0.01	2577	1.04 (0.98; 1.09)	0.16

Associations are expressed as odds ratios (OR) with 95% confidence intervals (CI) per 10 nmol/L increase in 25(OH)D3. Only fasting individuals with newly diagnosed glucose intolerance were included in the analyses.

* IFG versus normal glucose tolerance.

^†^ IGT versus normal glucose tolerance.

^‡^Type 2 diabetes versus normal glucose tolerance, IFG, IGT and IFG+IGT.

^§^Vitamin D or multivitamins.

## Discussion

Based on the existing literature, we expected to find an inverse linear or a non-linear (i.e. U-shaped or reverse J-shaped) association between 25(OH)D3 levels and markers of glucose homeostasis and a negative association with glucose intolerance [2, 11, 28–30]. Surprisingly, we found that increasing concentrations of serum 25(OH)D3 were associated with increasing fasting plasma glucose, 2hour plasma glucose and HbA_1c_ and decreasing beta-cell function after adjustment for sex, age, ancestry, BMI, smoking, physical activity, and supplementation with vitamin D or multivitamins. Furthermore, the odds of IFG and type 2 diabetes increased with increasing vitamin D. After additional adjustment for mercury concentrations the associations remained significant, except for type 2 diabetes. The finding of serum 25(OH)D3 levels measured in 1987 also being positively associated with HbA_1c_ gives further support to the observed association, although the estimates here were only adjusted for age and sex.

Interpreting these association patterns as a possible adverse effect of vitamin D on the underlying mechanisms of type 2 diabetes would be in contrast to the several lines of evidence supporting a favorable role for vitamin D in glucose homeostasis, including beneficial effects on pancreatic beta-cell function and insulin action [31]. Vitamin D appears to affect the insulin response to glucose stimulation directly by binding of the active form of vitamin D, 1,25(OH)2D, to the vitamin D receptors on beta-cells [31]. Furthermore, 1,25(OH)2D activates transcription of the human insulin receptor gene [32], stimulates the expression of insulin receptor, and enhances insulin-mediated glucose transport [33]. Insulin secretion is a calcium dependent process, and indirect effects of vitamin D may be mediated through its important role in regulating extracellular calcium and calcium flux through the beta-cell [31]. In support of these findings, prospective studies have shown a protective effect of high 25(OH)D concentrations on type 2 diabetes risk [13, 28]. However, extremely high concentrations of vitamin D, which may occur as a result of excessive supplemental intake, can be toxic and have adverse health effects [34]. The highest concentration of 25(OH)D3 measured in this study (255 nmol/L) is far below the level generally regarded as unfavorable or toxic (500 nmol/L) to human health [34]. We therefore find it most plausible that the observed positive associations with fasting- and 2hour plasma glucose and HbA_1c_ in our study are carried by one or more other factors associated with development of glucose intolerance and type 2 diabetes. Among such factors could be environmental contaminants. We adjusted for methyl mercury because we recently found a positive association between mercury and fasting- and 2hour plasma glucose, impaired fasting glucose and type 2 diabetes in the same study population [35]. However, even after adjustment for mercury, we found positive associations between 25(OH)D3 and glucose measures. Vitamin D is a strong marker of the intake of the traditional marine diet known to provide high levels of other types of environmental contaminants not accounted for here, and these might influence the association between vitamin D and type 2 diabetes. Furthermore, other micronutrients, such as phosphorus [36] and iron [37, 38], may affect the associations.

Alternatively, metabolism of vitamin D may play a role. In a study reporting an association between high concentrations of vitamin D and increased risk of prostate cancer [39] the authors suggested a role for 24-hydroxylase. This enzyme is stimulated by high concentrations of plasma and intracellular 25(OH)D3 to rapidly hydroxylate vitamin D metabolites on carbon 24 to produce either inactive 24,25(OH)D or degrade the active form of vitamin D, 1,25(OH)2D3, to the inactive 1,24,25-trihydroxyvitamin D3 metabolite. This will lower concentrations of intracellular 1,25(OH)2D3 [39, 40], which could then allow increased proliferation of malignant cells and hence an increased risk of prostate cancer. A similar 24-hydroxylase-based degradation mechanism in response to high vitamin D concentrations may apply in type 2 diabetes. Low concentrations of 1,25(OH)2D resulting from degradation in response to high 25(OH)D3 concentrations, could be speculated to provide insufficient stimulation of vitamin D receptors in beta-cells, resulting in impaired beta-cell function and impaired insulin secretion [31], which in turn may result in elevated blood glucose levels. Adverse effects of low vitamin D on beta-cell function has been documented previously [31]. Rapid degradation of 1,25(OH)2D by 24-hydroxylase may have been beneficial in former generations in order to avoid toxic vitamin D levels obtained from the traditional diet. Such rapid degradation may, however, be unfavourable among present-day Inuit relying primarily on imported dietary sources with lower content of vitamin D. The fact that a proportion of the study participants has a maintained high intake of traditional diet and a high serum 25(OH)D3 level, together with our finding of a significant decrease in beta-cell function, may support the above hypothesis.

Regardless of the underlying mechanisms behind the weak positive associations between 25(OH)D3 levels and glucose measures, these associations are considered of limited clinical importance. With a modest increase in fasting- and 2hour plasma glucose of 0.02 mmol/L and 0.05 mmol/L, respectively, and 0.39% increase in HbA_1c_ per 10 nmol/L increase in serum 25(OH)D3 concentration, the contribution of lifestyle related risk factors such as smoking, physical inactivity and a diet containing high levels of saturated fat and sugar may affect glucose homeostasis measures substantially more than vitamin D status. Moreover, a recent genome-wide association study among Inuit in Greenland has discovered a frequent variant of the gene TBC1D4 associated with type 2 diabetes, which has been estimated to account for more than 10 percent of all type 2 diabetes cases in Greenland [41]. Thus, genetic status is likely overruling the role of vitamin D in this population. This is further supported by the lack of an association between 25(OH)D3 and type 2 diabetes, and only a weak association with IFG, after adjustment for mercury. Our findings may thus support the indications from Mendelian randomization studies and randomized placebo-controlled trials that the association between 25(OH)D3 concentration and type 2 diabetes is unlikely to be causal (9, 10).

Serum 25(OH)D3 in 1987 and 2005–2010 was measured in blood samples stored for 3–26 years, which may introduce the risk of decay. However, higher contents of 25(OH)D3 in samples from 1987 than in 2005–2010 samples opposes this. Evaporation of the liquid part in the test vial and thereby higher 25(OH)D3 concentration in the remaining serum, which could be another concern, is unlikely to have occurred as sera were kept in vials with impervious lids and wrapped in foil until thawing. Previous research has documented that the concentration of 25(OH)D3 appears to be stable in serum despite long storage periods [42] and exaggerated conditions [43]. Thus, the reported serum 25(OH)D3 concentrations in samples from 1987 and 2005–2010 are likely to reflect the content at the time of sampling.

Strengths of the study included the use of serum 25(OH)D3 measurements from a large stratified random sample of Inuit in Greenland with clinical measures of glucose homeostasis and glucose intolerance. Furthermore, methyl mercury was measured, and detailed information on sociodemographic and lifestyle conditions was available from questionnaires. By using blood samples collected from a sub-sample of the IHIT study population in 1987 we were able to estimate associations between serum 25(OH)D3 and markers of glucose homeostasis and glucose intolerance in both 1987 and 2005–2010 allowing testing for robustness of association patterns. While 1987 levels of 25(OH)D3 supported a positive association with HbA_1c_, there was no association with fasting- and 2hour plasma glucose, beta-cell function, HOMA-IR, IFG, IGT and type 2 diabetes, and the size of the estimate and the wide confidence limits for the association with IFG indicated that the sample size was too small to detect a true association. Thus, the lack of an association with IFG may be due to reduced power when using the serum 25(OH)D3 measurements from 1987. To further investigate the unexpected finding of a positive association between 25(OH)D3 and HbA_1c_, and the indication of non-linearity, we used multiple regression with quadratic splines to visually investigate this relation. The graphical presentation supported a positive linear relation between 25(OH)D3 and HbA_1c_. To exclude the risk of reverse causality related to life style changes, only previously unknown diabetes and pre-diabetes cases were included in the study. Among limitations of the study were the determination of serum 25(OH)D3 based on one measurement in the IHIT study, which may have introduced measurement error, and over- and under-estimation of serum 25(OH)D3 status in summer and winter seasons may have occurred due to the fact that samples were not available from July, November and December. This is, however, unlikely to have affected the results. Another obvious limitation is the cross-sectional nature of the study which does not allow determination of cause-effect relationships between exposure and outcome variables.

In conclusion, this large investigation undertaken in a randomly selected adult Inuit population, found increasing serum 25(OH)D3 levels to be weakly associated with increased fasting- and 2hour plasma glucose and HbA_1c_, increased odds of IFG, and decreased beta-cell function. These findings are in contrast to some previous reports of favorable effects of vitamin D on beta-cell function and glucose homeostasis. We found no association between 25(OH)D3 levels and peripheral insulin sensitivity, HOMA-IR, IGT and type 2 diabetes. Overall, the results could not support an association between low vitamin D status and risk of type 2 diabetes in Greenland and emphasize the need to elucidate a causal relationship between vitamin D and glucose intolerance.
